# An Enhanced Three-Factor User Authentication Scheme Using Elliptic Curve Cryptosystem for Wireless Sensor Networks

**DOI:** 10.3390/s17122946

**Published:** 2017-12-19

**Authors:** Chenyu Wang, Guoai Xu, Jing Sun

**Affiliations:** New Research Activities Darparment, Beijing University of Posts and Telecommunications, Haidian District, Beijing 100876, China; wangchenyu@bupt.edu.cn (C.W.); sunjing@bupt.edu.cn (J.S.)

**Keywords:** user authentication, smart card, offline dictionary attack

## Abstract

As an essential part of Internet of Things (IoT), wireless sensor networks (WSNs) have touched every aspect of our lives, such as health monitoring, environmental monitoring and traffic monitoring. However, due to its openness, wireless sensor networks are vulnerable to various security threats. User authentication, as the first fundamental step to protect systems from various attacks, has attracted much attention. Numerous user authentication protocols armed with formal proof are springing up. Recently, two biometric-based schemes were proposed with confidence to be resistant to the known attacks including offline dictionary attack, impersonation attack and so on. However, after a scrutinization of these two schemes, we found them not secure enough as claimed, and then demonstrated that these schemes suffer from various attacks, such as offline dictionary attack, impersonation attack, no user anonymity, no forward secrecy, etc. Furthermore, we proposed an enhanced scheme to overcome the identified weaknesses, and proved its security via Burrows–Abadi–Needham (BAN) logic and the heuristic analysis. Finally, we compared our scheme with other related schemes, and the results showed the superiority of our scheme.

## 1. Introduction

With its strong self-organization, low-cost, resource-limited and data-centered, wireless sensor networks (WSNs) have been widely deployed in harsh environments such as military, industrial, transportation and even battlefields. Different to some systems such as the distributed architectures [1,2], there are three participants in WSNs. Each participant has different computational and storage power, and only the gateway can store the long-term key. Furthermore, most sensor nodes are distributed in an unattended environment, which means the sensor node is prone to be attacked. It also should be noted that the communications between users and sensor nodes are usually in an open channel, and the adversary can eavesdrop on or modify messages in the network. Therefore, the privacy and security of WSNs are always the thorny and vital issues. To deal with these security issues, it is a common practice to establish a security mechanism to share secret key between communicating parties and encrypt the date from remote parities. In this context, the remote user authentication protocol [3,4,5,6,7] with a session key is an essential security strategy for a secure and practical communication over an untrusted but complicated network. It guarantees that the communicating parties can verify the validity of each other and negotiate a session key for encrypting the future transmitted messages. The major challenge in designing an authentication protocol in WSNs is to balance the relationship between security, privacy and computational cost.

Generally, we authenticate a remote user from three aspects: what he knows, such as password; what he owns, such as a smart card; who he is, such as biometrics. A scheme using “X” aspects to verify the remote user is called “X-factor” authentication protocol. With the development of biotechnology and the increasing demands on security, three-factor (password + smart card + biometrics) user authentication scheme gets widely applied.

### 1.1. Related Works

In 2009, Das [8] introduced a password-based scheme with a smart card for WSNs; it then aroused an intense discussion and greatly promoted the development of user authentication in WSNs. Many researchers [4,9,10,11] identified the security pitfalls in Das’s scheme [8] (such as being prone to offline password guessing attack, impersonation attack and insider attack), and then proposed many enhanced versions. However, none of these schemes was secure enough to resist against various attacks or achieved low computational cost.

In 2011, Fan et al. [12] criticized the weakness of previous schemes and designed a new scheme with lightweight operations. With lower computational cost, their scheme seems quite suitable for a resources-limited environment such as WSNs. In 2012, Das et al. [13] proposed a new scheme which supports the dynamical addition of new nodes and only involves some lightweight operations. It has to be admitted that Das et al.’s scheme provides many desired attributes. Unfortunately, Wang et al. [14] identified that the two schemes both are vulnerable to many attacks: Fan et al.’s scheme [12] can neither achieve user anonymity, nor avoid smart card lost attack and insider attack, etc.; Das et al.’s scheme cannot resist against insider attack, smart card lost attack, etc.

In 2013, Xue et al. [15] introduced an efficient authentication scheme with admirable features and lightweight computational cost. However, it was revealed by Wang et al. [3] that this scheme fails to achieve user anonymity. Furthermore, Li et al. [16] demonstrated its vulnerability to offline dictionary attack, insider attack, stolen-verifier attack, etc., and proposed a new scheme which is still insecure against offline dictionary attack. In the same year, Li et al. [17] identified the weakness (not resistant to dictionary attack and session key disclosure attack, etc.) in Yeh et al.’s scheme [18].

In 2014, Choi et al. [19] showed that a previous scheme [20] suffers from sensor energy exhausting attack, offline password guessing attack and the session key attack, and then proposed a new scheme. After demonstrating the security flaws in Xue et al.’s scheme [15], Jiang et al. [21] also designed an improved one. However, both the scheme of Choi et al. [19] and Jiang et al. [21] were discovered as not being secure as claimed by Wu et al. [22].

In 2015, He et al. [23] described a temporal-credential-based scheme for WSNs, yet soon was pointed out subject to impersonation attack, smart card lost attack and tracking attack. In the same year, Chang et al. [24] proposed an enhanced dynamic identity authentication, once again, it was proved not secure against offline password guessing attack, user impersonation attack, etc. by Jung et al. [25] and Park et al. [26]. To strengthen the security of the scheme, Jung et al. [25] and Park et al. [26] both added the biological characteristic as a new factor and proposed a three-factor enhanced version. Furthermore, they both proved the security of their scheme formally, so they were confident in the security of their scheme.

### 1.2. Motivations and Contributions

When revisiting Jung et al.’s scheme [25] and Park et al.’s scheme [26], it was regretful to find that the two schemes are still not as secure as claimed, though they both are equipped with the complete formal proof, and furthermore, add a biometric factor into the scheme to improve the security of the previous scheme. Ridiculously, the improved two schemes that are armed with a biometric factor and a formal proof, even cannot provide the same level security assurance as the previous ones. We find them vulnerable to offline password guessing attack, impersonation attack, and no user anonymity, no forward security, etc.

In fact, it is pretty common that a scheme with formal security proof was found insecure. Though the user authentication in wireless sensor networks have been developed over almost ten years since Das [8] first proposed a two-factor scheme, there is not yet a secure and practical scheme. Even more alarming is the fact that many schemes violate some basic design principles that have been proposed. Such an unsatisfactory situation prompts us to design a secure but efficient scheme for wireless sensor networks. Furthermore, the common consensus on the system architecture, adversary model and security requirements should be reached. In conclusion, our contributions are as follows:

1. We depict the system architecture, adversary model and security requirements of wireless sensor networks. Though these factors are the basis of the authentication scheme, researchers usually ignore them.
2. We demonstrate that: (1) Jung et al.’s scheme cannot resist against offline password guessing attack, impersonation attack, and fails to achieve user anonymity and forward secrecy, etc.; (2) Park et al.’s scheme suffers from offline password guessing attack, and no user anonymity. Furthermore, we explain the inherent reason for these attacks.
3. We propose an improved scheme with various desirable attributes, and prove its security via BAN logic and heuristic analysis. Then, we compare our scheme with other related schemes. The results show the great advantage of our scheme.

### 1.3. Organization of the Paper

The remainder of this paper is organized as follows: we describe the system architecture and adversary model in Section 2, analyze Jung et al.’s scheme and Park et al.’s scheme in Section 3 and Section 4, respectively; in Section 5, we propose an enhanced scheme; the security and performance analysis are given in Section 6 and Section 7, respectively; and the conclusions are drawn in Section 8.

## 2. Preliminaries

This section introduces the preliminaries in the user authentication scheme including computational problems, system architecture, adversary model and security requirements.

### 2.1. Computational Problems

Given two large primes *p* and *q*, let $F_{p}$ be a finite field, $E/F_{p}$ be an elliptic curve over $F_{p}$, and G be a *q*-order subgroup of $E/F_{p}$. Then, for $\alpha ,\beta \in Z_{p}^{*}$ and a point *P* in G, we can define the discrete logarithm problem over the elliptic curve as follows:

1. Elliptic curve discrete logarithm problem: given (*P*, αP), it is impossible to compute α within polynomial time.
2. Elliptic curve computational Diffie–Hellman problem: given (αP, βP), it is impossible to compute αβP within polynomial time.

### 2.2. System Architecture

**Figure 1:**
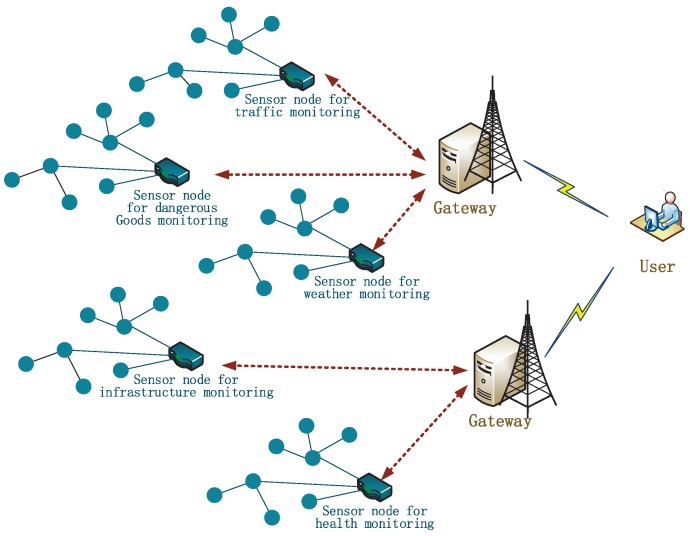
WSNs system architecture.

Wireless sensor networks (as shown in Figure 1) attract worldwide attention with the prevalence of Internet of Things (IoT). Generally, people may be more familiar with distributed systems, which involve two participants: a set of users and a single server, while there are three participants in the user authentication of WSNs: a number of sensor nodes, a gateway node and a set of users. In a wireless sensor network, there are tens to thousands of sensor nodes that are deployed in a particular area. They work together to collect the data from physical world and have limited computing and storage power. Furthermore, they are usually left in an unattended environment, so the adversary can easily capture them to acquire secret parameters. The gateway node acts like a registration center. In WSNs, an authentication protocol usually consists of four basic phases: registration, login, verification, and password update. Sometimes, the dynamic node addition phase is suggested for meeting the demand on increasing new sensor nodes. In the registration phase, users and sensor nodes submit their personal information to the gateway, then the gateway will issue users a smart card with some sensitive parameters physically (face to face or via the mail), and distribute a shared secret key to sensor nodes. When a user wants to access a sensor node, he/she can initialize an access request to the gateway in the login phase. After checking the legitimacy of the user, the gateway informs the corresponding sensor node about the request. Then, the user and the sensor node verify the legitimacy of each other via (or not) the gateway and negotiate a session key in the verification phase. The user can change the password in the password update phase. In addition, the new sensor nodes can join the network in the dynamic node addition phase.

### 2.3. Adversary Models

When considering cryptanalysis of the user authentication schemes in WSNs, the adversary A is also supposed to have the following capacities:

1. A can fully control the open communication channel, i.e., A can modify, intercept, delete, and resend the eavesdropped on messages over an open channel [9,27].
2. A can enumerate all the items in $D_{pw}*D_{id}$ in polynomial time, where $D_{pw}$ and $D_{id}$ denote the password space and the identity space, respectively [28,29].
3. When evaluating forward secrecy, A can get the long-term secret key [28,30].
4. A can acquire the password of a legitimate user by a malicious card reader, or get the parameters in the smart card, but cannot achieve both [28,30].
5. A can get the data in sensor nodes for they are usually left unattended [3,31].
6. A can get the past session keys [30].
7. A can get the user’s biometrics [29,32].

The capacity of acquiring biometrics is the most controversial. Many researchers view it as a quite strong factor that cannot be broken. However, this is impractical. For example, the adversary can at least get the biometrics via a malicious terminal. Moreover, unlike the password that may change with the different applications, the biometrics is unique to every particular person. Thus, the adversary can collect one’s biometrics via any biometric-based terminal. This indicates that the adversary can acquire the password and biometrics both, or the smart card and the biometrics both. Furthermore, this hypothesis has been accepted in many schemes, such as [29,32,33].

It should be noted that: a secure three-factor authentication scheme should guarantee that the breaking of any two of the three factors will not affect the other one, and the system is still secure.

### 2.4. Security Requirements

Understanding the security requirements of the user authentication is a fundamental step to analyze or design a protocol. Thus, we summarize the security requirements of user authentication in the wireless sensor network:

**S1**  Mutual authentication. It is an essential requirement in all authentication schemes. It requires the participants to authenticate each other [34,35].
**S2**  User anonymity. It is a privacy protection requirement for individual users, not directly related to system security. Many systems have such a requirement including distributed system [36]. While the privacy protection in wireless sensor networks is more severe, since the information among sensor nodes (usually unreliable) is transmitted in a way of broadcasting. Protecting user anonymity is to stop A from computing the user’s identity or linking the transcript to a same user. Note that such a requirement is not applied to the gateway, but to sensor nodes for they are untrusted.
**S3**  Key agreement. It is also an essential requirement in most authentication schemes. The session key is used to encrypt the further communications to achieve confidentiality.
**S4**  Forward secrecy. It is for the final collapse of the whole system, and it requires that the previous communications will be secure, even the system collapses (usually refers to the adversary that owns the long-term key of the system).
**S5**  User friendly. It is an additional requirement to improve the user experience with the development of the network. A user friendly scheme usually includes: let the user $U_{i}$ select the password freely, and change it locally [30]; when $U_{i}$ finds the smart card insecure, let he/she revoke it and re-register to the system with original identity.
**S6**  No stolen-verifier attack. It is a requirement related to the security of the whole system (so as the following attacks), which requires that the verifier table does not expose any sensitive information for A to impersonate the participants or learn/control the session key.
**S7**  No insider attack. It requires that the participants cannot get any sensitive information, which may provoke an attack.
**S8**  No dictionary attack. It requires that A cannot conduct a brute force attack.
**S9**  No replay attack. It stops A from conducting an attack via replaying the history message, which requires that the participants can check the freshness and validity of the received message.
**S10**  No parallel session attack. This requirement is a bit similar to the replay attack, but it considers a condition where A conducts an attack via initiating multi-session simultaneously.
**S11**  No de-synchronization attack. The synchronization attack in wireless sensor networks is more destructive than that in traditional networks, since a gateway may connect even hundreds of sensor nodes. It requires that the parameters among corresponding participants are consistent.
**S12**  No impersonation attack. It is a very important requirement in authentication, which requires that the outside adversary (inside adversary has been considered in insider attack) will not be able to impersonate any participants. A scheme resistant to impersonation attack requires that the participants verify whether the corresponding communication party is a counterfeit one. Note that: the occasion where A performs a user impersonation attack using the password from a dictionary attack is not included—such an attack belongs to dictionary attack.
**S13**  No known key attack. It is an attack related to the session key, which requires A, who knows that the current session key cannot compute the keys in others.

## 3. Cryptanalysis of Jung et al.’s Scheme

In 2017, Jung et al. [25] demonstrated several attacks against Chang et al.’s [24] two-factor user authentication scheme in WSNs. To improve the security and practicability of the scheme, they devised an enhanced one over Chang et al.’s scheme [24] by “employing biometrics information with the biohashing technique". They proved their scheme secure to various attacks such as offline dictionary attack using the Burrows-Abadi-Needham (BAN) logic. However, as we will show in this section, Jung et al.’s scheme still suffers from offline dictionary attack, impersonation attack, etc., which is even less secure than the previous one. For convenience of illustration, some notations are listed in Table 1.

### 3.1. A Brief Review of Jung et al.’s Scheme

In this section, we review Jung et al.’s scheme [25] briefly, their scheme consists of four phases: registration, login, verification and password change. The password change phase was omitted, since it has little relevance to this work.

#### 3.1.1. Registration Phase

In Jung et al.’s paper, there is only a user registration phase as follows:

1. $U_{i}\Rightarrow GW$: $\left\{TID_{i},HPW_{i}\right\}$, where $TID_{i}$ = $h(ID_{i}||u)$, $HPW_{i}$ = $h(PW_{i}||H\left(BIO_{i}\right))$ and *u* is a random number chosen by the user $U_{i}$.
2. $GW\Rightarrow U_{i}$: a smart card containing $\left\{A_{i},E_{i},C_{i},h\left(\cdot \right),H\left(\cdot \right)\right\}$, where $HID_{i}$ = $h(TID_{i}\left||x\right)⊕HPW_{i}$, $A_{i}$ = $h(HPW_{i}||TID_{i})⊕\;HID_{i}$, $E_{i}$ = $h(HPW_{i}||HID_{i})$, $C_{i}$ = $HID_{i}⊕x$.
3. $U_{i}$ stores $D_{i}$ in the smart card, where $D_{i}$ = $u⊕H(BIO_{i})$.

However, according to the paper, the sensor node $S_{j}$ preserves a private key $X_{s_{j}}$. So we deduce that the sensor node registration phase was missed. For the integrity, we add it as below:

1. $S_{j}\Rightarrow GW$: $\left\{SID_{j}\right\}$.
2. $GW\Rightarrow S_{j}$: $X_{s_{j}}$ = $h(SID_{j}||x)$.
3. $S_{j}$ stores $X_{s_{j}}$ as a secret key.

#### 3.1.2. Login Phase and Verification Phase

1. $U_{i}\to GW$: $\left\{DID_{i},M_{U_{i},G},C_{i},T_{1}\right\}$. $U_{i}$ inputs the $ID_{i}$ and $PW_{i}$, and his biometrics $BIO_{i}$; then, the smart card computes:
  $\begin{array}{c} HPW_{i}^{*}=h(PW_{i}||H\left(BIO_{i}\right)),\\ u=D_{i}⊕H\left(BIO_{i}\right),\\ TID_{i}=h(ID_{i}||u),\\ HID_{i}^{*}=A_{i}⊕h(HPW_{i}^{*}||TID_{i}^{*}),\\ E_{i}=h(HPW_{i}^{*}||HID_{i}^{*}). \end{array}$
  Finally, the card checks $E_{i}^{*}$ ?= $E_{i}$. If it is equal, the card computes $DID_{i}$ = $TID_{i}⊕HID_{i}^{*}$ and $M_{U_{i},G}$ = $h(TID_{i}||HPW_{i}^{*}||HID_{i}^{*}||T_{1})$, and sends $\left\{DID_{i},M_{U_{i},G},C_{i},T_{1}\right\}$ to GW. Otherwise, it ends the session.
2. $GW\to S_{j}$: $\left\{DID_{i},M_{G,S_{j}},M_{j},T_{2}\right\}$. GW first checks the freshness of $T_{1}$, then computes:
  $\begin{array}{c} TID_{i}^{*}=DID_{i}⊕C_{i}⊕x,\\ HID_{i}=C_{i}⊕x\\ HPW_{i}^{*}=HID_{i}⊕h(TID_{i}^{*}||x),\\ M_{U_{i},G}^{*}=h(TID_{i}^{*}||HPW_{i}^{*}||HID_{i}\left|\right|T_{1}), \end{array}$
  and further tests $M_{U_{i},G}^{*}$?= $M_{U_{i},G}$. If the condition is not satisfied, GW rejects the request. Otherwise, it computes $X_{s_{j}}$ = $h(SID_{j}||x)$, $M_{j}$ = $R⊕X_{s_{j}}$, SK = $f(DID_{i},R)$ and $M_{G,S_{j}}$ = $h(DID_{i}||SID_{j}||X_{s_{j}}||SK||T_{2})$, and sends $\left\{DID_{i},M_{G,S_{j}},M_{j},T_{2}\right\}$ to $S_{j}$.
3. $S_{j}\to GW$: $\left\{M_{S_{j},G},T_{3}\right\}$. $S_{j}$ first checks $T_{2}$, and computes:
  $\begin{array}{c} R^{*}=M_{j}⊕X_{s_{j}},\\ SK^{*}=f\left(DID_{i},R^{*}\right),\\ M_{G,S_{j}}^{*}=h(DID_{i}||SID_{j}\left|\right|X_{s_{j}}||SK^{*}\left|\right|T_{2}). \end{array}$
  If $M_{G,S_{j}}^{*}$ = $M_{G,S_{j}}$, $S_{j}$ further computes $k_{j}$ = $h(X_{s_{j}}||T_{3})$, $M_{S_{j},G}$ = $h(k_{j}||X_{s_{j}}||SK^{*}||T_{3})$, and sends $\left\{M_{S_{j},G},T_{3}\right\}$ to the GW. Otherwise, it exits the session.
4. $GW\to U_{i}$: $\left\{k_{i},M_{G,U_{i}},T_{4}\right\}$. GW first checks $T_{3}$, and computes:
  $\begin{array}{c} k_{j}^{*}=h(X_{s_{j}}\left|\right|T_{3}),\\ M_{S_{j},G}^{*}=h(k_{j}^{*}\left|\right|X_{s_{j}}\left||SK|\right|T_{3}). \end{array}$
  If $M_{S_{j},G}^{*}$ = $M_{S_{j},G}$, GW further computes $k_{i}$ = $R⊕h(TID_{i}^{*}||x)$, $M_{G,U_{i}}$ = $h(SK||k_{i}||||T_{4})$, and sends $\left\{k_{i},M_{G,U_{i}},T_{4}\right\}$ to the GW. Otherwise, it exits the session.
5. $U_{i}$ first checks $T_{4}$, and computes $R^{*}$ = $k_{i}⊕HPW_{i}⊕HID_{i}^{*}$, $SK^{*}$ = $f(DID_{i},R^{*})$ and $M_{G,U_{i}}^{*}$ = $h(SK^{*}||k_{i}||T_{4})$. If $M_{G,U_{i}}^{*}$ = $M_{G,U_{i}}$, $U_{i}$ believes the legitimacy of GW and the authentication phase ends successfully. Otherwise, the authentication fails.

### 3.2. Security Flaws in Jung et al.’s Scheme

Jung et al. [25] criticized that Chang et al.’s scheme [24] fails to resist against offline password guessing attack and the session key attack. Thus, they add a new factor to enhance the security of the previous two-factor scheme, and formed a three-factor one. Despite armed with the biometrics factor and provable security proof, their scheme suffers from the same (even more serious) security issues.

#### 3.2.1. Offline Dictionary Attack

Offline dictionary attack is exactly what most schemes suffer from and also the major security requirement of a user authentication protocol. Jung et al. [25] showed that Chang et al.’s scheme [24] cannot resist against this attack once the adversary breaches the victim’s smart card and eavesdrops on the message from the open channel. Unfortunately, as we show below, the same attack also works for Jung et al.’s own scheme. In addition, Jung et al.’s scheme is vulnerable to other kinds of offline dictionary attacks with less attack cost.

According to the adversary capabilities mentioned in Section 2.3, it is natural to suppose that the adversary A somehow possessed $U_{i}$’s smart card and then revealed the message $\left\{A_{i},E_{i},C_{i},D_{i}\right\}$ in it; acquired $U_{i}$’s biometric $BIO_{i}$ by a malicious terminal or other ways; and intercepted transcripts $\left\{DID_{i},M_{U_{i},G},C_{i},T_{1}\right\}$ via the public channel. Then, A can obtain $U_{i}$’s password $PW_{i}$ as follows:

1. Guesses the value of $PW_{i}$ to be $PW_{i}^{*}$ and $ID_{i}$ to be $ID_{i}^{*}$ from the dictionary space $D_{pw}$ and $D_{id}$, respectively. In fact, according to Wang et al. [28], once an adversary picks the victim’s ($U_{i}$) smart card, it is easy to learn the corresponding identity $ID_{i}$ of the user $U_{i}$.
2. Computes $HPW_{i}^{*}$ = $h(PW_{i}^{*}||H\left(BIO_{i}\right))$.
3. Computes *u* = $D_{i}⊕H\left(BIO_{i}\right)$, where $D_{i}$ is from the smart card.
4. Computes $TID_{i}^{*}$ = $h(ID_{i}^{*}||u)$.
5. Computes $HID_{i}^{*}$ = $A_{i}⊕h(HPW_{i}^{*}||TID_{i}^{*})$, where $A_{i}$ is from the card.
6. Computes $M_{U_{i},G}^{*}$ = $h(TID_{i}^{*}||HPW_{i}^{*}||HID_{i}^{*}||T_{1})$, where $T_{1}$ is from the public channel.
7. Verifies the correctness of $PWi^{*}$ and $ID_{i}^{*}$ by checking if the computed $M_{U_{i},G}^{*}$ is equal to the intercepted $M_{U_{i},G}$.
8. Repeats Steps 1–7 of this procedure until the correct value of $PW_{i}$ and $ID_{i}$ is found.

The time complexity of the above attack is $O(|D_{pw}|*|D_{id}|*\left(5T_{H}\right))$. $T_{H}$ is the running time for hash computation. $|D_{pw}|$ denotes the number of passwords in $D_{pw}$. $|D_{pw}|$ and $|D_{id}|$ are very limited, generally $|D_{id}\left|<\right|D_{pw}|<10^{6}$ [30,37], so the above attack is quite efficient.

Besides the above kind of offline dictionary attack, Jung et al.’s scheme still suffers from another kind of offline dictionary attack where the adversary A obtained the victim’s smart card and the biometrics $BIO_{i}$. Then, A can conduct another offline dictionary attack as follows (Steps 1–5 are the same with the above attack, so they are omitted):

1. *Step* 6. Computes $E_{i}$ = $h(HPW_{i}^{*}||HID_{i}^{*})$, where $A_{i}$ is from the card.
2. *Step* 7. Verifies the correctness of $PW_{i}^{*}$ and $ID_{i}^{*}$ by checking if $E_{i}^{*}$ ?= $E_{i}$.
3. *Step* 8. Repeats Steps 1–7 of this procedure until the correct value of $PW_{i}$ and $ID_{i}$ is found.

The time complexity of the attack is the same as the former attack. Actually, these two attack strategies are not new, and many researchers [32,36,38,39,40] have captured these two attack scenarios to break numerous schemes. However, these kinds of adversaries are still rampant.

**Remark** **1.** As we mentioned before, a true three-factor authentication scheme should ensure that even if any two of the three factors are compromised, the other factor cannot be breached and the entire system is still secure. Obviously, this protocol is intrinsically not a three-factor protocol. It indicates that the biometric factor is not a master key to settle the problem in user authentication. On the contrary, a scheme armed with biometrics factor may even cannot provide the same security level as a two-factor authentication. The way to add more factors into the authentication protocol is not the essential way to design a more secure protocol.

In the scheme of Jung et al. [25], the obstacles to compute the verification value $M_{U_{i},G}$ for an adversary A is the $PW_{i}$ and the $ID_{i}$, so A can guess the value of the $PW_{i}$ and the $ID_{i}$, then verify the guessed value by comparing the computed $M_{U_{i},G}^{*}$ with the intercepted $M_{U_{i},G}$. This is exactly the essential reason for the former kind of offline dictionary attack. Similarly, $E_{i}$ is also the fuse of the latter kind of attack. However, the function of the two parameters is quite different: the $M_{U_{i},G}$ is the key of the GW to authenticate $U_{i}$, while the $E_{i}$ contributes to changing the password locally and detecting incorrect input timely. Therefore, the $M_{U_{i},G}$ is indispensable to an authentication protocol, and the $E_{i}$ conduces to improve the usability of a scheme. Furthermore, the “public-key principle” is necessary to resist the former attack [41]; and a way of “honeywords” + “fuzzy-verifiers” is suggested by Wang et al. [30] to deal with the latter attack.

#### 3.2.2. Impersonation Attack

Suppose an adversary A was also a legal user $U_{a}$, then he could get the secret key *x* as follows:

1. Computes *u* = $D_{a}⊕H\left(BIO_{a}\right)$, where $D_{a}$ is from the smart card.
2. Computes $TID_{a}$ = $h(ID_{a}||u)$.
3. Computes $HPW_{a}$ = $h(PW_{a}||H\left(BIO_{i}\right))$.
4. Computes $HID_{a}$ = $A_{a}⊕h(HPW_{a}||TID_{a})$, where $A_{a}$ is from the card.
5. Computes *x* = $C_{a}⊕HID_{a}$, where $C_{a}$ is from the card.

Obviously, the time complexity of the above attack is $O(5T_{H}+3T_{R})$, where $T_{R}$ is the running time for exclusive-or operation. With the secret *x*, A has the same capacity as the GW, thus A can impersonate as the GW or the $S_{j}$; this indicates that the security of the whole system collapsed.

Actually, not only can an insider legal user carry out such an attack, but also an adversary who has gotten the PW and ID of any users by “offline dictionary attack” can also perform such an attack. The $C_{i}$ ($C_{i}$ = $HID_{i}⊕x$) is the fundamental reason for such an attack. To a legitimate user who knows the $HID_{i}$, the secret key *x* is actually exposed. Therefore, the only “XOR” operation on *x* is a risky behavior which is far from enough to protect such an significant parameter.

#### 3.2.3. User Anonymity

User anonymity is of great significance to privacy protection. It requires that the adversary can neither confirm who transmits the messages nor recognize whether the messages come from the same user. In wireless sensor networks, numerous sensor nodes are deployed in an unattended environment. In addition, the information is transmitted in a way of broadcasting. Therefore, user anonymity in WSNs is an essential requirement. However, in Jung et al.’s scheme [25], user-specific parameters $DID_{i}$ and $C_{i}$ are transmitted via an open channel. Thus, following $DID_{i}$ or $C_{i}$, the adversary A identifies the transmitted messages with the $DID_{i}$ and $C_{i}$ from a large amount of messages in the open channel, and links them to the user $U_{i}$. Then, for the purpose of marketing or even other terrible attempts, the A can learn the user $U_{i}$’s habits, such as the time to initiate an access request, the kinds of sensor nodes to visit. Therefore, Jung et al’s scheme fails to achieve user anonymity.

#### 3.2.4. Forward Secrecy

Forward secrecy requires that even if the long-term secret key was exposed, the adversary still cannot compute the previous session key. In other words, when the long-term key is compromised, the protocol cannot promise the security of further communications, but it can guarantee the security of the previous communication. Forward secrecy is the last umbrella of system security, but Jung et al.’s scheme fails to achieve it.

Supposing that an adversary A got the secret key *x* and intercepted the parameters $DID_{i}$ and $M_{j}$ in the channel, A could perform an attack to get the previous session key as follows:

1. Computes $X_{s_{j}}$ = $h(SID_{j}||x)$.
2. Computes *R* = $M_{j}⊕X_{s_{j}}$, where $M_{j}$ is from the open channel.
3. Computes SK = $f(DID_{i},R)$, where $DID_{i}$ is from the open channel.

**Remark** **2.** In this scheme, the session key consists of a fixed parameter $DID_{i}$ and a random number R from GW. As $DID_{i}$ is exposed to an open channel, the only challenge in computing the session key is the value of R. On one hand, the sensor node $S_{j}$ has to know R to form the session key. This means that the $S_{j}$ is capable of computing R. On the other hand, $S_{j}$’s special or only secret parameter is $X_{s_{j}},$ where $X_{s_{j}}$ = $h(SID_{j}||x)$. Thus, once acquiring $X_{s_{j}}$ and the transmitted message in an open channel, anyone can compute the session key. Therefore, when an adversary learns the long-term key x, he/she has the same capability as the $S_{j}$. Of course, he/she can compute the correct session key. In fact, it is a more secure way to set up the session key with the security mechanism of challenge-response for the two sides of communication. Anyway, all this corroborates that a protocol without any exponentiation operations conducted on the server side cannot achieve forward secrecy [41].

## 4. Cryptanalysis of Park et al.’s Scheme

Similar to Jung et al., Park et al. [26] also criticized Chang et al.’s scheme [24], and improved this two-factor scheme into a three-factor one. They claimed their new scheme overcomes the weaknesses in [24], and proved the security of the scheme via BAN logic. Unfortunately, we once again found this scheme also insecure: no resistance to two kinds of offline dictionary attacks and no user anonymity.

### 4.1. A Brief Review of Park et al.’s Scheme

This section describes Park et al.’s scheme [26] briefly.

#### 4.1.1. Registration Phase

Note that the senor node registration phase is the same with Jung et al.’s [25], so it is omitted here.

1. $U_{i}\Rightarrow GW$: $\left\{ID_{i},HPW_{i}\right\}$, where $\left(R_{i},P_{i}\right)$ = $Gen(BIO_{i})$, $HPW_{i}$ = $h(PW_{i}||R_{i})$.
2. $GW\Rightarrow U_{i}$: a smart card containing $\left\{A_{i},B_{i},C_{i},TID_{i},h\left(\cdot \right)\right\}$, where $HID_{i}$ = $h(ID_{i}||x)$, $X_{s_{i}}$ = $h(HID_{i}||x)$, $A_{i}$ = $h(HPW_{i}\left|\right|X_{s_{i}})⊕HID_{i}$, $B_{i}$ = $h(HPW_{i}||X_{s_{i}})$, $C_{i}$ = $X_{s_{i}}⊕h(ID_{i}\left|\right|HPW_{i}$. Furthermore, GW stores $\left(TID_{i},TID_{i}^{\circ }\right)$ in database, and $TID_{i}$ is a random number, $TID_{i}^{\circ }$ is initialized to NULL.
3. $U_{i}$ inputs $P_{i}$ into the smart card. Note that, in Park et al.’s scheme [26], this step is not mentioned. But, according to the scheme, this step is necessary. We speculate it is missed.

#### 4.1.2. Login Phase and Verification Phase

1. $U_{i}\to GW$: $\left\{DID_{i},X_{i},M_{U_{i},G},T_{i},TID_{i}\right\}$. $U_{i}$ inputs the $ID_{i}$ and $PW_{i}$, and the biometrics $BIO_{i}$, and then the smart card computes:
  $\begin{array}{c} R_{i}^{*}=Rep\left(BIO_{i},P_{i}\right),\\ HPW_{i}^{*}=h(PW_{i}\left|\right|R_{i}^{*}),\\ X_{s_{i}}^{*}=C_{i}⊕h(ID_{i}||HPW_{i}^{*}),\\ B_{i}^{*}=h\left(HPW_{i}^{*}⊕X_{s_{i}}^{*}\right). \end{array}$
  If $B_{i}^{*}$ == $B_{i}$, the card selects a random number $\alpha \in Z_{p}^{*}$, and computes $X_{i}$ = αP, $k_{i}$ = $h(X_{s_{i}}^{*}||T_{i})$, $DID_{i}$ = $h(HPW_{i}\left|\right|X_{s_{i}}^{*})⊕k_{i}$ and $M_{U_{i},G}$ = $h(A_{i}||X_{s_{i}}^{*}||X_{i}||T_{i})$, and sends $\left\{DID_{i},X_{i},M_{U_{i},G},T_{i},TID_{i}\right\}$ to GW. Otherwise, it ends the session.
2. $GW\to S_{j}$: $\left\{DID_{i},M_{G,S_{j}},X_{i},T_{G}\right\}$. GW first checks $T_{i}$, then gets $HID_{i}$ and computes:
  $\begin{array}{c} X_{s_{i}}^{\prime }=h(HID_{i}||x),\\ k_{i}^{\prime }=h(X_{s_{i}}^{\prime }\left|\right|T_{i}). \end{array}$
  If $M_{U_{i},G}$≠$h(h\left(DID_{i}⊕k_{i}^{\prime }⊕HID_{i}\right)||X_{s_{i}}^{\prime }||X_{i}||T_{i})$, GW rejects the request. Otherwise, it computes $X_{s_{j}}^{\prime }$ = $h(SID_{j}||x)$, $M_{G,S_{j}}$ = $h(DID_{i}||SID_{j}||X_{s_{j}}^{\prime }||X_{i}||T_{G})$, and sends $\left\{DID_{i},M_{G,S_{j}},X_{i},T_{G}\right\}$ to $S_{j}$.
3. $S_{j}\to GW$: $\left\{M_{S_{j},G},Y_{j},T_{j}\right\}$. $S_{j}$ first checks $T_{G}$, and if $M_{G,S_{j}}$≠$h(DID_{i}||SID_{j}||X_{s_{j}}||X_{i}||T_{G})$, rejects it. Otherwise, $S_{j}$ chooses $b\in Z_{p}^{*}$ and computes:
  $\begin{array}{c} Y_{j}=\beta P,\\ k_{j}=h(X_{s_{j}}\left|\right|T_{j}),\\ Z_{i}=M_{G,S_{j}}⊕k_{j}\\ SK_{j}=h(DID_{i}\left|\right|k_{j}||\beta X_{i}),\\ M_{S_{j},G}=h(Z_{i}\left|\right|X_{s_{j}}^{*}\left|\right|X_{i}\left|\right|Y_{j}\left|\right|T_{j}), \end{array}$
  and sends $\left\{M_{S_{j},G},Y_{j},T_{j}\right\}$ to the GW.
4. $GW\to U_{i}$: $\left\{e_{i},M_{G,U_{i}},Y_{j},T_{G}^{\prime }\right\}$. GW first checks $T_{j}$, and computes $k_{j}^{\prime }$ = $h(X_{s_{j}}^{\prime }||T)j)$, $Z_{i}^{\prime }$ = $M_{G,S_{j}}⊕k_{j}^{\prime }$, if $M_{S_{j},G}$ == $h(Z_{i}^{\prime }||X_{s_{j}}^{\prime }||X_{i}||X_{j}||T_{j})$, GW further computes:
  $\begin{array}{c} e_{i}=k_{j}⊕h\left(k_{i}\right),\\ M_{G,U_{i}}=h(DID_{i}\left|\right|M_{U_{i},G}\left|\right|k_{j}^{\prime }\left|\right|X_{s_{i}}^{\prime }\left|\right|X_{i}\left|\right|Y_{j}\left|\right|T_{G}^{\prime }),\\ TID_{i_{new}}=h(HID_{i}\left|\right|T_{i}), \end{array}$
  and updates $\left(TID_{i},TID_{i}^{\circ }\right)$ as $\left(TID_{i_{new}},TID_{i}\right)$, then sends $\left\{e_{i},M_{G,U_{i}},Y_{i},T_{G}^{\prime }\right\}$ to the GW. Otherwise, it exits the session.
5. $U_{i}$ checks $T_{G}^{\prime }$, and computes $k_{j}^{*}$ = $e_{i}⊕h\left(k_{i}\right)$, if $M_{G,U_{i}}$ == $h(DID_{i}||M_{U_{i},G}||k_{j}^{÷}||X_{s_{i}}^{*}||X_{i}||Y_{j}||T_{G}^{\prime })$, computes SK = $h(DID_{i}||k_{j}^{*}||\alpha Y_{j})$, and updates $TID_{i}$ as $h(HID_{i}||T_{i})$. Otherwise, it exits the session.

### 4.2. Security Flaws in Park et al.’s Scheme

Compared with Jung et al. [25], Park et al. [26] deployed an elliptic curve cryptosystem trying to achieve user anonymity and resist against offline dictionary attack. Though Wang et al. [3,41] pointed out that a public key algorithm is necessary to achieve user anonymity and offline dictionary attack, it does not mean that, once the public key algorithm is added, the system will be secure. Deploying the public key algorithm requires some skills, and we will propose a sound scheme as an example to explain such skills in Section 5. In this section, we proved that Park et al.’s scheme suffers from many attacks, including offline dictionary attack and no user anonymity.

#### 4.2.1. Offline Dictionary Attack

Suppose the adversary A got the message $\left\{A_{i},B_{i},C_{i},P_{i},TID_{i}\right\}$ in the card; and also acquired $U_{i}$’s biometrics $BIO_{i}$ in addition to intercepted transcripts $\left\{DID_{i},X_{i},M_{U_{i},G},T_{i},TID_{i}\right\}$. Then, A conducts an offline dictionary attack as follows:

1. Guesses $PW_{i}$ to be $PW_{i}^{*}$ and $ID_{i}$ to be $ID_{i}^{*}$.
2. Computes $R_{i}^{*}$ = $Rep(BIO_{i},P_{i})$, where $P_{i}$ is from the smart card.
3. Computes $HPW_{i}^{*}$ = $h(PW_{i}^{*}||R_{i}^{*})$.
4. Computes $X_{s_{i}}^{*}$ = $C_{i}⊕h(ID_{i}^{*}||HPW_{i}^{*})$.
5. Computes $M_{U_{i},G}^{*}$ = $h(A_{i}||X_{s_{i}}^{*}||X_{i}||T_{i})$, where $A_{i}$ is from the card, $X_{i}$ and $T_{i}$ are from the channel.
6. Verifies the correctness of $PWi^{*}$ and $ID_{i}^{*}$ by checking whether $M_{U_{i},G}^{*}$ == $M_{U_{i},G}$.
7. Repeats Step 1–7 of this procedure until the correct value of $PW_{i}$ and $ID_{i}$ is found.

The time complexity of the above attack is $O(|D_{pw}|*|D_{id}|*\left(3T_{H}+T_{RE}\right))$. $T_{RE}$ is the running time of fuzzy extraction computation. Thus, the above attack is quite efficient.

Similar to the analysis in Section 3.2.1, the adversary can also select $B_{i}$ as the verification to test the guessed value of $PW_{i}^{*}$ and $ID_{i}^{*}$.

#### 4.2.2. User Anonymity

Park et al. [26] attempted to update some parameters to provide user anonymity. However, such a method is not as desirable as they expected. On one hand, the gateway has to update the database in every session, which is efficient; on the other hand, if the adversary A acquires the verifier table $\left\{TID_{i},TID_{i}^{\circ },HID_{i})\right\}$, and intercepts $\left\{DID_{i},X_{i},M_{U_{i},G},T_{i},TID_{i}\right\}$, then A can find the $TID_{i}$ from the verifier table and get the corresponding $HID_{i}$. Now, A can compute the $TID_{i_{new}}$ in the next session as $h(HID_{i}||T_{i})$. Thus, A can link the login request to the same user who has ever used $TID_{i}$ via computing $TID_{i_{new}}$ in every session. Thus, Park et al.’s scheme cannot achieve user anonymity.

## 5. Proposed Scheme

**Figure 2:**
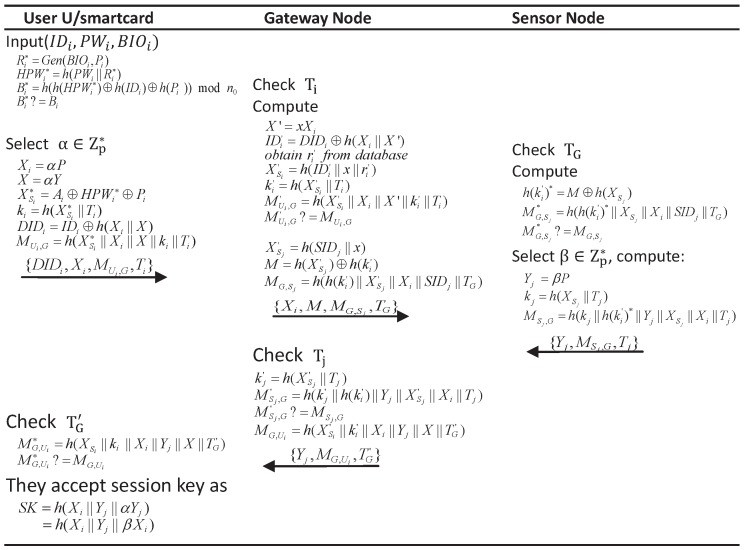
Proposed scheme.

In this section, we proposed a new enhanced scheme (as shown in Figure 2) which not only provides some desirable attributes but also can resist against the known attacks. Furthermore, we improve the scheme from the following aspects:

1. based on Wang et al. [3,41], we apply a public key algorithm for resisting against offline dictionary attack via the verification from the open channel. In such an attack, as we analyzed above, the key solution is about the way to construct the verification parameter between the user and the gateway node. Once the verification parameter consists of a “challenge” that is deployed a public key algorithm, a trap door will be built. Therefore only the one who owns the corresponding secret key can compute the correct “challenge” (i.e., *X* in our scheme). In Park et al.’s scheme, though a public key algorithm is deployed, it is not used to construct a “challenge” for authentication. More specifically, all the parameters in the verification $M_{U_{i},G}$ (=$h(A_{i}||X_{S_{i}}||X_{i}||T_{i})$) can be computed with the static or open knowledge in the user side and the open channel, so A can compute all parameters ($A_{i}$,$X_{S_{i}}$,$X_{i}$,$T_{i}$) with guessed password and then use $M_{U_{i},G}$ to verify the guessed value. While, in our new scheme, a “challenge” *X* is built. Besides the static or open knowledge on the user side, A has to know the dynamic α or the long-term key to compute *X*, and thus fails to conduct such an attack;
2. as introduced in Section 3.2.1, we use “honeywords” + “fuzzy-verifiers” to resist against offline dictionary attack via verification from the smart card [30,42];
3. we do not protect user anonymity via updating parameters as Park et al., but deploy a dynamic identity technique via a public key algorithm [3].

The details of our scheme is described as follows:

### 5.1. Registration Phase

The registration phase to the sensor node is similar to Jung et. al. [25] and Park et. al. [26], so it is omitted. When a new user wants to be a legitimate user of the system, then he/she may submit his/her personal information on the gateway to initiate a user registration phase as follows:

1. $U_{i}\Rightarrow GW$: $\left\{ID_{i},HPW_{i}\right\}$, where $\left(R_{i},P_{i}\right)$ = $Gen(BIO_{i})$, $HPW_{i}$ = $h(PW_{i}||R_{i})$.
2. $GW\Rightarrow U_{i}$: a smart card containing $\left\{A_{i},B_{i},n_{0},Y,P\right\}$, where $X_{s_{i}}$ = $h(ID_{i}||x||r_{i})$ ($r_{i}$ is a random number), $A_{i}$ = $X_{s_{i}}⊕HPW_{i}⊕P_{i}$, $B_{i}$ = $h(h\left(HPW_{i}\right)⊕h\left(ID_{i}\right)⊕h\left(P_{i}\right))$ mod $n_{0}$, *Y* = xP. Furthermore, GW stores $\left(ID_{i},r_{i},Honey\_List\right)$ in database, and Honey_List is supposed to count the number of failing in user login phase and it is initialized to NULL. Once its value is bigger than the predetermined threshold, the corresponding smart card will be discarded till the user re-registers.
3. $U_{i}$ inputs $P_{i}$ into the smart card.

### 5.2. Login Phase and Verification Phase

After being legitimated, the user $U_{i}$ can login to the system with the password, identity and biometrics, and get authenticated via exchanging information with the corresponding communication parties. Finally, after finishing the authentication successfully, the user and the sensor node will build a session key to protect the security of the subsequent communications.

1. $U_{i}\to GW$: $\left\{DID_{i},X_{i},M_{U_{i},G},T_{i}\right\}$. $U_{i}$ inputs his/her identity $ID_{i}$, password $PW_{i}$, and biometrics $BIO_{i}$; then, the smart card computes:
  $\begin{array}{c} R_{i}^{*}=Rep\left(BIO_{i},P_{i}\right),\\ HPW_{i}^{*}=h(PW_{i}\left|\right|R_{i}^{*}),\\ B_{i}^{*}=h\left(h\left(HPW_{i}^{*}\right)⊕h\left(ID_{i}\right)⊕h\left(P_{i}\right)\right)\;mod\;n_{0}. \end{array}$
  If $B_{i}^{*}$ == $B_{i}$, the card accepts the user, and selects a random number $\alpha \in Z_{p}^{*}$, computes:
  $\begin{array}{c} X_{i}=\alpha P,\\ X=\alpha Y,\\ X_{s_{i}}^{*}=A_{i}⊕HPW_{i}^{*}⊕P_{i},\\ k_{i}=h(X_{s_{i}}^{*}\left|\right|T_{i}),\\ DID_{i}=ID_{i}⊕h(X_{i}||X),\\ M_{U_{i},G}=h(X_{s_{i}}^{*}\left|\right|X_{i}\left||X|\right|k_{i}\left|\right|T_{i}), \end{array}$
  and then sends $\left\{DID_{i},X_{i},M_{U_{i},G},T_{i}\right\}$ as a login request to GW. Otherwise, it ends the session.
2. $GW\to S_{j}$: $\left\{X_{i},M,M_{G,S_{j}},T_{G}\right\}$. GW first checks the freshness of $T_{i}$, computes:
  $\begin{array}{c} X^{\prime }=xX_{i},\\ ID_{i}^{\prime }=DID_{i}⊕h(X_{i}\left|\right|X^{\prime }), \end{array}$
  and then finds $r_{i}^{\prime }$ and Hony_List via $ID_{i}^{\prime }$. If Hony_List≥ the preset value (for example 10), the GW thinks this smart card has been suspended and rejects the request. Otherwise, GW computes $X_{s_{i}}^{\prime }$ = $h(ID_{i}^{\prime }||x||r_{i}^{\prime })$, $k_{i}^{\prime }$ = $h(X_{s_{i}}^{\prime }||T_{i})$. If $M_{U_{i},G}$≠$h(X_{s_{i}}^{\prime }||X_{i}||X^{\prime }||k_{i}^{\prime }||T_{i})$, GW rejects the request and sets Hony_List = Hony_List+1. Once Hony_List is bigger than the preset value, the corresponding smart card is suspended. Otherwise, it computes:
  $\begin{array}{c} X_{s_{j}}^{\prime }=h(SID_{j}||x),\\ M=h\left(X_{s_{j}}^{\prime }\right)⊕h\left(k_{i}^{\prime }\right),\\ M_{G,S_{j}}=h(h\left(k_{i}^{\prime }\right)\left|\right|X_{s_{j}}^{\prime }\left|\right|X_{i}||SID_{j}\left|\right|T_{G}), \end{array}$
  and sends $\left\{X_{i},M,M_{G,S_{j}},T_{G}\right\}$ to $S_{j}$ to conveys $U_{i}$’s request.
3. $S_{j}\to GW$: $\left\{Y_{j},M_{S_{j},G},T_{j}\right\}$. $S_{j}$ first checks the valid of $T_{G}$, and computes $h{\left(k_{i}^{\prime }\right)}^{*}$ = $M⊕h(X_{s_{j}})$. If $M_{G,S_{j}}^{*}$≠$h(h{\left(k_{i}^{\prime }\right)}^{*}||X_{s_{j}}||X_{i}||SID_{j}||T_{G})$, $S_{j}$ does not believe GW and rejects the session. Otherwise, $S_{j}$ chooses $\beta \in Z_{p}^{*}$ and computes:
  $\begin{array}{c} Y_{j}=\beta P,\\ k_{j}=h(X_{s_{j}}\left|\right|T_{j}),\\ SK_{j}=h(X_{i}\left|\right|Y_{j}||\beta X_{i}),\\ M_{S_{j},G}=h(k_{j}||h{\left(k_{i}^{\prime }\right)}^{*}\left|\right|Y_{j}\left|\right|X_{s_{j}}\left|\right|X_{i}\left|\right|T_{j}), \end{array}$
  and sends $\left\{Y_{j},M_{S_{j},G},T_{j}\right\}$ to GW.
4. $GW\to U_{i}$: $\left\{Y_{j},M_{G,U_{i}},T_{G}^{\prime }\right\}$. GW first checks $T_{j}$. Then, it computes $k_{j}^{\prime }$ = $h(X_{s_{j}}^{\prime }||T_{j})$, and if $M_{S_{j},G}$ == $h(k_{j}||h\left(k_{i}^{\prime }\right)||Y_{j}||X_{s_{j}}^{\prime }||X_{i}||T_{j})$, GW further computes $M_{G,U_{i}}$ = $h((X_{s_{i}}^{\prime }\left|\right|k_{i}^{\prime }\left|\right|X_{i}\left|\right|Y_{j}\left||X|\right|T_{G}^{\prime })$, and then sends $\left\{Y_{j},M_{G,U_{i}},T_{G}^{\prime }\right\}$ to $U_{i}$ to transmit $S_{j}$’s responds. Otherwise, it exits the session.
5. $U_{i}$ first checks $T_{G}^{\prime }$, and if $M_{G,U_{i}}$ == $h((X_{s_{i}}\left|\right|k_{i}\left|\right|X_{i}\left|\right|Y_{j}\left||X|\right|T_{G}^{\prime })$, $U_{i}$ authenticates the GW, and computes $SK_{i}$ = $h(X_{i}||Y_{j}||\alpha Y_{j})$ to finish the authentication successfully. Otherwise, the authentication fails.

### 5.3. Password Change Phase

Once the user wants to change password for security consideration, he/she can achieve it through the following steps:

1. $U_{i}$ inputs $ID_{i}$, $PW_{i}$ and new password $PW_{i}^{new}$.
2. The card computes:
  $\begin{array}{c} R_{i}^{*}=Rep\left(BIO_{i},P_{i}\right),\\ HPW_{i}^{*}=h(PW_{i}\left|\right|R_{i}^{*}),\\ B_{i}^{*}=h\left(h\left(HPW_{i}^{*}\right)⊕h\left(ID_{i}\right)⊕h\left(P_{i}\right)\right)\;mod\;n_{0}. \end{array}$
  If $B_{i}^{*}\neq B_{i}$, the card does not permit $U_{i}$ to change the password. Otherwise, it further computes:
  $\begin{array}{c} HPW_{i}^{new}=h(PW_{i}^{new}\left|\right|R_{i}^{*}),\\ B_{i}^{new}=h\left(h\left(HPW_{i}^{new}\right)⊕h\left(ID_{i}\right)⊕h\left(P_{i}\right)\right)\;mod\;n_{0},\\ A_{i}^{new}=A_{i}⊕HPW_{i}^{*}⊕HPW_{i}^{new}, \end{array}$
  and finally replaces $A_{i},B_{i}$ with $A_{i}^{new},B_{i}^{new}$.

### 5.4. Revocation Phase

Revocation phase, as the emergency response strategy, is of great significance to the security of the system. It provides an efficient way to protect the account from being abused. When the user finds his/her smart card breached, he/she can revoke the smart card as follows:

1. $U_{i}$ firstly get authenticated by the card as the way to the step 1 in Section 5.2.
2. $U_{i}⟶GW$: $\left\{DID_{i},X_{i},M_{U_{i},G},T_{i},revoke\_request\right\}$. As described in Section 5.2, the smart card computes $DID_{i}$,$X_{i}$,$M_{U_{i},G}$ and sends $\left\{DID_{i},X_{i},M_{U_{i},G},T_{i},revoke\_request\right\}$ to the gateway.
3. After receiving the revocation request from $U_{i}$, GW first verifies $U_{i}$. If GW authenticates $U_{i}$ successfully, it sets Honey_List to a big number, which is bigger than the preset value. Then, the smart card will be revoked, and nobody can login to the system with the card unless $U_{i}$ re-register. Otherwise, GW rejects the request.

### 5.5. Re-Register Phase

If a user $U_{i}$ with correct password and identity is still rejected by $S_{j}$, then can re-register as follows:

1. $U_{i}⟹GW$: $\left\{ID_{i},HWR_{i},P_{i},re-register\right\}$.
2. Firstly, GW looks for $ID_{i}$ from User-list, checks whether Honey_List≥ the preset value. If so, GW believes the card is suspended, then performs the corresponding steps in Section 5.1.

## 6. Security Analysis

To prove the security of our scheme, we analyze it from two aspects: a formal way using the Burrows–Abadi–Needham (BAN) logic [43]; a informal/heuristic way. Through the formal way, we prove our scheme achieves four basic security goals. These goals ensure that the user and the sensor node are mutual trust, and they both compute the session key successfully; furthermore, the session keys computed by them are equal. Through the informal/heuristic way, we prove that our scheme not only satisfies many desired attributes such as user anonymity and forward security, but also is resistant to various attacks such as offline dictionary attack, impersonation attack, and de-synchronized attack.

### 6.1. Formal Analysis Based on BAN Logic

The BAN logic [43] is a simple and efficient way to analyze the design logic and security of a protocol. It has a set of particular notions (shown in Table 2) to depict the logic of the protocol. We will prove the security of our scheme according to its notions and processes.

In BAN logic, the goals of our authentication scheme are defined as:

- Goal 1: $U_{i}|\equiv S_{j}|\equiv \left(U_{i}\overset{SK}{↔}S_{j}\right)$.
- Goal 2: $U_{i}|\equiv \left(U_{i}\overset{SK}{↔}S_{j}\right)$.
- Goal 3: $S_{j}|\equiv U_{i}|\equiv \left(U_{i}\overset{SK}{↔}S_{j}\right)$.
- Goal 4: $S_{j}|\equiv \left(U_{i}\overset{SK}{↔}S_{j}\right)$.

According to the proof steps in BAN logic, we re-describe our scheme into an idealized form:

- $M_{1}$: $U_{i}\to GW$: ${\langle X_{i},k_{i},T_{i},DID_{i},U_{i}\overset{X}{↔}GW\rangle }_{U_{i}\overset{X_{s_{i}}}{↔}GW}$.
- $M_{2}$: $GW\to S_{j}$: ${\langle X_{i},h\left(k_{i}\right),SID_{j},T_{G}\rangle }_{S_{j}\overset{X_{s_{j}}}{↔}GW}$.
- $M_{3}$: $S_{j}\to GW$: ${\langle X_{j},k_{j},h\left(k_{i}\right),T_{j}\rangle }_{S_{j}\overset{X_{s_{j}}}{↔}GW}$.
- $M_{4}$: $GW\to U_{i}$: ${\langle X_{j},k_{i},X,T_{G}^{\prime }\rangle }_{U_{i}\overset{X_{s_{i}}}{↔}GW}$.

Then, some assumptions are defined as follows:

- $H_{1}$: $U_{i}|\equiv ♯\left(T_{G}^{\prime }\right)$.
- $H_{2}$: $S_{j}|\equiv ♯\left(T_{G}\right)$.
- $H_{3}$: $GW|\equiv ♯(T_{i})$.
- $H_{4}$: $GW|\equiv ♯(T_{j})$.
- $H_{5}$: $GW|\equiv S_{j}\overset{X_{s_{j}}}{↔}GW$.
- $H_{6}$: $S_{j}|\equiv S_{j}\overset{X_{s_{j}}}{↔}GW$.
- $H_{7}$: $GW|\equiv U_{i}\overset{X_{s_{i}}}{↔}GW$.
- $H_{8}$: $U_{i}|\equiv GW\overset{X_{s_{i}}}{↔}RC$.
- $H_{9}$: $U_{i}|\equiv S_{j}|\Rightarrow U_{i}\overset{SK}{↔}S_{j}$.
- $H_{10}$: $S_{j}|\equiv U_{i}|\Rightarrow U_{i}\overset{SK}{↔}S_{j}$.

Based on the definition above, we perform the BAN logic proof as follows: 

**From** $M_{1}$, it is easy to get $S_{1}$: $GW◃{\langle X_{i},k_{i},T_{i},DID_{i},U_{i}\overset{X}{↔}GW\rangle }_{X_{s_{i}}}.$

Then, according to $H_{7}$, $S_{1}$, RULE(1), we get $S_{2}$: $GW|\equiv U_{i}|∼\langle X_{i},k_{i},T_{i},DID_{i},U_{i}\overset{X}{↔}GW\rangle .$

According to $H_{3}$ and RULE(4), we get $S_{3}$: $GW|\equiv ♯\langle X_{i},k_{i},T_{i},DID_{i},U_{i}\overset{X}{↔}GW\rangle .$

In addition, according to $S_{2}$, $S_{3}$ and RULE(2), $S_{4}$: $GW|\equiv U_{i}|\equiv \langle X_{i},k_{i},T_{i},DID_{i},U_{i}\overset{X}{↔}GW\rangle$

**From** $M_{2}$, it is easy to get $S_{5}$: $S_{j}◃{\langle X_{i},h\left(k_{i}\right),SID_{j},T_{G}\rangle }_{X_{s_{j}}}.$

Then, according to $H_{7}$, $S_{1}$, RULE(1), we get $S_{6}$: $S_{j}|\equiv GW|∼\langle X_{i},h\left(k_{i}\right),SID_{j},T_{G}\rangle .$

According to $H_{3}$ and RULE(4), we get $S_{7}$: $S_{j}|\equiv ♯\langle X_{i},h\left(k_{i}\right),SID_{j},T_{G}\rangle .$

In addition, according to $S_{2}$, $S_{3}$ and RULE(2), we get $S_{8}$: $S_{j}|\equiv GW|\equiv \langle X_{i},h\left(k_{i}\right),SID_{j},T_{G}\rangle .$

**From** $M_{3}$, it is easy to get $S_{9}$: $GW◃{\langle X_{j},k_{j},h\left(k_{i}\right),T_{j}\rangle }_{X_{s_{j}}}.$

Then, according to $H_{7}$, $S_{1}$, RULE(1), we get $S_{10}$: $GW|\equiv S_{j}|∼\langle X_{j},k_{j},h\left(k_{i}\right),T_{j}\rangle .$

According to $H_{3}$ and RULE(4), we get $S_{11}$: $GW|\equiv ♯\langle X_{j},k_{j},h\left(k_{i}\right),T_{j}\rangle .$

In addition, according to $S_{2}$, $S_{3}$ and RULE(2), we get $S_{12}$: $GW|\equiv S_{j}|\equiv \langle X_{j},k_{j},h\left(k_{i}\right),T_{j}\rangle .$

**From** $M_{4}$, it is easy to get $S_{13}$: $U_{i}◃{\langle X_{j},k_{i},X,T_{G}^{\prime }\rangle }_{X_{s_{i}}}.$

Then, according to $H_{7}$, $S_{1}$, RULE(1), we get $S_{14}$: $U_{i}|\equiv GW|∼\langle X_{j},k_{i},X,T_{G}^{\prime }\rangle .$

According to $H_{3}$ and RULE(4), we get $S_{15}$: $U_{i}|\equiv ♯\langle X_{j},k_{i},X,T_{G}^{\prime }\rangle .$

In addition, according to $S_{2}$, $S_{3}$ and RULE(2), we get $S_{16}$: $U_{i}|\equiv GW|\equiv \langle X_{j},k_{i},X,T_{G}^{\prime }\rangle .$

As SK = $h(X_{i}||X_{j}||\alpha X_{j})$, and combining $S_{12}$, $S_{16}$, we get: $S_{17}$: $U_{i}|\equiv S_{j}|\equiv U_{i}\overset{SK}{↔}S_{j}$ (**Goal 1**).

Similarly, as SK = $h(X_{i}||X_{j}||\beta X_{i})$, with $S_{4}$, $S_{8}$, we get: $S_{18}$: $S_{j}|\equiv U_{i}|\equiv U_{i}\overset{SK}{↔}S_{j}$ (**Goal 3**).

Finally, according to $H_{2}$, $S_{17}$ and RULE(3), we get: $S_{19}$: $U_{i}|\equiv \left(U_{i}\overset{SK}{↔}S_{j}\right)$ (**Goal 2**).

In addition, according to $H_{10}$, $S_{18}$ and RULE(3), we get: $S_{20}$: $S_{j}|\equiv \left(U_{i}\overset{SK}{↔}S_{j}\right)$ (**Goal 4**). 

Therefore, we prove our scheme achieves Goals 1–4 successfully. In other words, our scheme promises that $U_{i}$ and $S_{j}$ have been authenticated mutually, and they further compute and share the same session key SK.

### 6.2. Informal Analysis

The heuristic way plays an important role in testing the security of the user authentication protocol. It makes up for the defects of formal proofs in some security requirements. For example, the formal proofs cannot capture user anonymity and user friendly problems. Therefore, in this section, we apply the heuristic method to prove the security of our scheme.

#### 6.2.1. Mutual Authentication

In step 2 and step 5 of Section 5.2, the gateway node and the user authenticate each other via their shared secret parameter $X_{s_{i}}$ and *X*. On the user side, only with the correct password, biometrics, and the corresponding smart card can $U_{i}$ compute $X_{s_{i}}$, so the gateway can authenticate $U_{i}$ via this parameter. On the gateway node, after receiving $X_{i}$, only the one with the long-term key *x*, can compute *X*, so the user authenticate GW via *X*.

In step 3 and step 4 of Section 5.2, the gateway node and the sensor node authenticate each other via $X_{s_{j}}$. If an adversary wants to compute $X_{s_{j}}$, then he/she has to guess the long-term key *x*, and the probability of such an event can be ignored.

Therefore, the user and the sensor node have authenticated the gateway, and the gateway has also authenticated them. Furthermore, from the authentication relationship among the three parties, equivalently, the user and the sensor node get authenticated with each other. All in all, our scheme achieves mutual authentication well.

#### 6.2.2. User Anonymity

In our scheme, $ID_{i}$ is concealed in $DID_{i}$, which is changed with *X* in every session. To get $ID_{i}$, A has to compute *X*, which means that A without α or *x* has to solve the elliptic curve discrete logarithm problem. As we introduced in Section 2.1, such a problem cannot be solved in polynomial time. Thus, in our scheme, the user identity is not only well protected, but also untraceable.

Furthermore, note that an obvious difference in user anonymity between the wireless sensor network and the distributed network is about whether the user identity can be known by other participants. In a distributed network, there are only two participants: the user and the server. In such a condition, the user identity can be known by the server to build a session key. While in the wireless sensor network, there are three participants: the user, the gateway node and the sensor node. The gateway node acts as a register center and is protected well, so it can know the user identity. While the sensor node is usually deployed in an unattended environment, it is of high possibility to be controlled by the adversary. Thus, the user identity should not be exposed to it. In addition, our scheme achieves such a goal: the user identity is not transmitted to the sensor node.

#### 6.2.3. Forward Secrecy

The session key SK = $h(X_{i}||Y_{j}||\beta X_{i})$ = $h(X_{i}||Y_{j}||\alpha Y_{j})$. The key parameter is $\beta X_{i}$ or $\alpha Y_{j}$. If an adversary A intercepts the message in an open channel, acquires the secret key *x*, then A knows $X_{i}$ and $X_{j}$. Thus, A needs to compute $\beta X_{i}$ or $\alpha Y_{j}$. However, computing $\beta X_{i}$ or $\alpha Y_{j}$ for A is equivalent to solving the Elliptic curve discrete logarithm problem, and it is bound to fail. Therefore, A cannot compute SK, and our scheme achieves forward secrecy.

#### 6.2.4. Offline Dictionary Attack

A sound three-factor user authentication scheme should ensure that even if A gets any two of the three factors, he/she cannot break the system. In our scheme, if A gets the password and biometrics, he/she still cannot compute $X_{s_{i}}$ to construct a valid login request; if A gets the password and the smart card, he/she can neither compute $X_{s_{i}}$ nor guess the biometrics, thus also fails to perform an attack; if A gets the smart card and biometrics, then A may conduct an offline dictionary attack by using $M_{u_{i},G}$ or $B_{i}$ as the verification parameter to check the correctness of the guessed value.

If A uses $B_{i}$, then he/she may make the offline dictionary attack as follows: guesses $ID_{i}$ and $PW_{i}$ to be $ID_{i}^{*}$ and $PW_{i}^{*}$, respectively, computes $R_{i}^{*}$ = $Rep(BIO_{i},P_{i})$, $HPW_{i}^{*}$ = $h(PW_{i}^{*}||R_{i}^{*})$, then verifies $ID_{i}^{*}$ and $PW_{i}^{*}$ by checking $B_{i}$ ?= $h(h\left(HPW_{i}^{*}\right)⊕h\left(ID_{i}\right)⊕h\left(P_{i}\right))$ mod $n_{0}$. However, even A gets a pair of $\left\{ID_{i}^{*},PW_{i}^{*}\right\}$ such that $B_{i}$ == $h(h\left(HPW_{i}^{*}\right)⊕h\left(ID_{i}\right)⊕h\left(P_{i}\right))$ mod $n_{0}$, he/she may not find the correct $ID_{i}$ and $PW_{i}$, for there are $|D_{pw}\left|*\right|D_{id}|\backslash n_{0}\approx 2^{32}$ candidates of $\left\{ID_{i},PW_{i}\right\}$ pair (where $n_{0}=2^{8}$ and $|D_{pw}\left|=\right|D_{id}|=2^{6}$) [30]. Thus, A then has to test the $\left\{ID_{i}^{*},PW_{i}^{*}\right\}$ via sending the login request to the gateway node, and once the number of login failures exceeds the preset value, the smart card will be suspended and the attack fails.

If A uses $M_{u_{i},G}$, then he/she can compute $HPW_{i}^{*}$ as above, and further compute $X_{s_{i}}^{*}$ = $A_{i}⊕HPW_{i}^{*}⊕P_{i}$, $k_{i}$ = $h(X_{s_{i}}^{*}||T_{i})$, $DID_{i}$ = $ID_{i}^{*}⊕h(X_{i}\left||X\right)$. However, A cannot compute *X*, as we explained in Section 6.2.2, and thus fails to finish such an attack.

In conclusion, our scheme is resistant to dictionary attack.

#### 6.2.5. Privileged Insider Attack

In our scheme, the user submits $\left\{ID_{i},HPW_{i},P_{i}\right\}$ to the gateway node. The password is well protected by a long-term number $R_{i}$, so GW cannot learn any useful information from it. Therefore, our scheme is secure against privileged insider attack.

#### 6.2.6. Verifier-Stolen Attack

The verifier table stored in GW does not expose sensitive messages; even if an adversary acquires the table, he/she cannot make any attack. Thus, our scheme is resistant to verifier-stolen attack.

#### 6.2.7. Replay Attack

The timestamp is used to prevent replay attack. On the one hand, if A replays the history message directly, the corresponding communication party will find it via checking the freshness of the timestamp. On the other hand, if A tries to forge the message in the open channel, such as $\left\{DID_{i},X_{i},M_{U_{i},G},T_{i}\right\}$, then he/she has to know $X_{s_{i}}$. However, to compute $X_{s_{i}}$, it is asked that A has to know *x* or $U_{i}$’s password, biometrics and smart card, which is impossible. Similarly, A also cannot replay or construct other message flows.

## 7. Performance Analysis

To better evaluate our scheme, we make a comparison among the related schemes for wireless sensor networks [25,26,29,44]. From Table 3, it is obvious that our scheme is more competitive than other schemes: our scheme achieves all the security requirements while others [25,26,29,44] all have some attributes that fail to satisfy more or less; the computation of our scheme is similar or slightly high to that of other schemes. Furthermore, achieving all the security requirements is more significant to an authentication scheme, and it is not advisable to sacrifice security for efficiency.

## 8. Conclusions

In this paper, we first introduced the system architecture of wireless sensor networks. Based on this, we summarized the adversary model and security requirements in such a special environment. Secondly, we identified the security flaws in two recent three-factor authentication schemes, and analyzed the inherent reasons for those flaws. Thirdly, we proposed an enhanced scheme resistant to various attack and with many desirable attributes. Then, we proved the security of our scheme via BAN logic and the heuristic analysis. Furthermore, the comparison with other related schemes showed the great advantage of our scheme. Finally, with the development of technology, Internet of Things and Internet of Vehicles will become more and more integrated into our daily life, and the ensuing security problems will become more and more prominent. Therefore, in the future, we will focus on identifying the security requirements and designing a secure but practical protocol in the authentication of these new application scenarios.

References

## Figures and Tables

**Table 1:** Notations and abbreviations.

Symbol	Description
$U_{i}$	ith user
GW	the gateway node
$S_{j}$	jth sensor node
A	malicious attacker
${\mathrm{ID}}_{i}$	identity of user $U_{i}$
$PW_{i}$	password of user $U_{i}$
$BIO_{i}$	biometrics of user $U_{i}$
$P_{j}$	the shared secret key between GW and $S_{j}$
x,y	the secret key of remote server GW
⊕	the bitwise exclusive OR (XOR) operation
∥	the string concatenation operation
$H(BIO_{i})$	collision free one-way hash function to the biometrics
h(·)	collision free one-way hash function
$Gen(BIO_{i})$	one part of fuzzy extraction function, output a biometric key $R_{i}$ and a helper string $P_{i}$
$Rep(BIO_{i},P_{i})$	one part of fuzzy extraction function, output the biometric key $R_{i}$ in $Gen(BIO_{i})$
→	a insecure channel
⇒	a secure channel

**Table 2:** Notations in BAN logic.

P∣≡X	*P* believes *X*, i.e., the principal *P* believes the statement *X* is true.
P◃X	*P* sees *X*, i.e., the principal *P* receives a message that contains *X*.
P∣⇒X	*P* has jurisdiction over *X*, i.e., the principal *P* can generate or compute *X*.
P∣∼X	*P* said *X*, i.e., the principal *P* has sent a message containing *X*.
♯(X)	*X* is fresh, i.e., *X* is sent in a message only at the current run of the protocol, it is usually a timestamp or a random number.
$P\overset{K}{↔}Q$	*K* is the shared key for *P* and *Q*.
$P\overset{Y}{⇌}Q$	*Y* is the secret known only to *P* and *Q* or some principals trusted by them.
${\langle X\rangle }_{Y}$	*X* combined with *Y*, and *Y* is usually a secret.
${\left\{X\right\}}_{K}$	*X* encrypted with *K*.
$\frac{P|\equiv P\overset{K}{↔}Q,P◃{\left\{X\right\}}_{K}}{P|\equiv Q|∼X}$ or $\frac{P|\equiv P\overset{Y}{⇌}Q,P◃{\langle X\rangle }_{Y}}{P|\equiv Q|∼X}$	RULE(1): the message-meaning rule.
	This rule will be used in the proving process.
$\frac{P|\equiv ♯(X),P|\equiv Q|∼X}{P|\equiv Q|\equiv X}$	RULE(2): the nonce-verification rule.
	This rule will be used in the proving process.
$\frac{P|\equiv Q|\Rightarrow X,P|\equiv Q|\equiv X}{P|\equiv X}$	RULE(3): the jurisdiction rule.
	This rule will be used in the proving process.
$\frac{P|\equiv ♯(X)}{P|\equiv ♯(X,Y)}$	RULE(4): the freshness-conjuncatenation rule.
	This rule will be used in the proving process.

**Table 3:** Performance comparison among relevant schemes in wireless sensor networks.

	Computation Overhead			Communication Cost			The Proposed Evaluation Criteria												
	Login (ms)	Auth. (ms)		Login	Auth.		S1	S2	S3	S4	S5	S6	S7	S8	S9	S10	S11	S12	S13
Amin et al. [44]	$8T_{H}\approx 0.055$	$25T_{H}\approx 0.017$		768 bits	1536 bits		√	×	√	×	√	√	√	×	√	√	√	√	√
Jiang et al. [29]	$T_{M}$+$6T_{H}\approx 1.2$	$T_{M}$+$19T_{H}\approx 1.2$		1408 bits	1280 bits		√	×	√	×	√	√	√	×	√	√	√	√	√
Jung et al. [25]	$5T_{H}\approx 0.0035$	$14T_{H}\approx 0.097$		512 bits	1024 bits		√	×	√	×	×	√	√	×	√	√	√	×	√
Park et al. [26]	$T_{E}$+$6T_{H}\approx 0.51$	$3T_{E}$+$18T_{H}\approx 1.5$		1536 bits	4096bits		√	×	√	√	×	×	√	×	√	√	√	√	√
Our scheme	$2T_{E}$+$8T_{H}\approx 1.0$	$4T_{E}$+$18T_{H}\approx 2.0$		1408 bits	3968 bits		√	√	√	√	√	√	√	√	√	√	√	√	√

$T_{M}$ is the time of modular exponentiation operation, $T_{E}$ is the time of scalar multiplication on elliptic curve, $T_{H}$ is the time of hash computation, $T_{M}\gg T_{E}\gg T_{H}$ (according to Wang et al. [28], $T_{M}\approx 1.169$ ms, $T_{E}\approx 0.508$ ms, $T_{H}\approx 0.693$μs) and the lightweight operation such as “XOR” and “||” can be ignored. Let $n_{0}$ be 32-bit long; Let $ID_{i}$, $PW_{i}$, h(∗), output of symmetric encryption, timestamp, random numbers be 128-bit long; Let *p*, *g*, *y* be 1024-bit long. √ means the property is satisfied; × means the property is not satisfied.

## References

[1] Pecori R., Veltri L. (2016). 3AKEP: Triple-authenticated key exchange protocol for peer-to-peer VoIP applications. Comput. Commun..

[2] Pecori R. A comparison analysis of trust-adaptive approaches to deliver signed public keys in P2P systems. Proceedings of the 7th International Conference on New Technologies, Mobility and Security (NTMS).

[3] Wang D., Wang P. (2014). On the anonymity of two-factor authentication schemes for wireless sensor networks: Attacks, principle and solutions. Comput. Netw..

[4] Khan M.K., Alghathbar K. (2010). Cryptanalysis and security improvements of “two-factor user authentication in wireless sensor networks”. Sensors.

[5] Kumar P., Choudhury J.A., Sain M., Lee S.G., Lee H.J. (2011). RUASN: A robust user authentication framework for wireless sensor networks. Sensors.

[6] Ling C., Lee C., Yang C., Hwang M. (2017). A secure and efficient one-time password authentication scheme for WSN. Int. J. Netw. Secur..

[7] Chen C.T., Lee C.C. (2013). A two-factor authentication scheme with anonymity for multi-server environments. Secur. Commun. Netw..

[8] Das M.L. (2009). Two-factor user authentication in wireless sensor networks. IEEE Trans. Wirel. Commun..

[9] Lee C., Li C., Chen S. (2011). Two attacks on a two-factor user authentication in wireless sensor networks. Parallel Process. Lett..

[10] Kumar P., Gurtov A., Ylianttila M., Lee S., Lee H. (2013). A strong authentication scheme with user privacy for wireless sensor networks. ETRI J..

[11] Sun D., Li J., Feng Z., Cao Z., Xu G. (2013). On the security and improvement of a two-factor user authentication scheme in wireless sensor networks. Pers. Ubiquitous Comput..

[12] Fan R., He D.P.X.P.L. (2011). An efficient and dos-resistant user authentication scheme for two-tiered wireless sensor networks. J. Zhejiang Univ. Sci. C.

[13] Das A.K., Sharma P., Chatterjee S., Sing J.K. (2012). A dynamic password-based user authentication scheme for hierarchical wireless sensor networks. J. Netw. Comput. Appl..

[14] Wang D., Wang P. (2014). Understanding security failures of two-factor authentication schemes for real-time applications in hierarchical wireless sensor networks. Ad Hoc Netw..

[15] Xue K., Ma C., Hong P., Ding R. (2013). A temporal-credential-based mutual authentication and key agreement scheme for wireless sensor networks. J. Netw. Comput. Appl..

[16] Li C.T., Weng C.Y., Lee C.C. (2013). An advanced temporal credential-based security scheme with mutual authentication and key agreement for wireless sensor networks. Sensors.

[17] Li C.T., Lee C.C., Lee C.W. (2013). An improved tTwo-factor user authentication protocol for wireless sensor networks using elliptic curve cryptography. Sens. Lett..

[18] Hsiu-Lien Y., Chen T.H., Liu P.C., Tai-Hoo K., Wei H.W. (2011). A secured authentication protocol for wireless sensor networks using elliptic curves cryptography. Sensors.

[19] Choi Y., Lee D., Kim J., Nam J., Won D. (2014). Security enhanced user authentication protocol for wireless sensor networks using elliptic curves cryptography. Sensors.

[20] Shi W., Gong P. (2013). A new user authentication protocol for wireless sensor networks using elliptic curves cryptography. Int. J. Distrib. Sens. Netw..

[21] Jiang Q., Ma J., Lu X., Tian Y. (2015). An efficient two-factor user authentication scheme with unlinkability for wireless sensor networks. Peer-to-Peer Netw. Appl..

[22] Wu F., Xu L., Kumari S., Li X. (2017). A new and secure authentication scheme for wireless sensor networks with formal proof. Peer-to-Peer Netw. Appl..

[23] He D., Kumar N., Chilamkurti N. (2015). A secure temporal-credential-based mutual authentication and key agreement scheme with pseudo identity for wireless sensor networks. Inf. Sci. Int. J..

[24] Chang I., Lee T., Lin T., Liu C. (2015). Enhanced two-factor authentication and key agreement using dynamic identities in wireless sensor networks. Sensors.

[25] Jung J., Moon J., Lee D., Won D. (2017). Efficient and security enhanced anonymous authentication with key agreement scheme in wireless sensor networks. Sensors.

[26] Park Y., Park Y. (2016). Three-factor user authentication and key agreement using elliptic curve cryptosystem in wireless sensor networks. Sensors.

[27] Huang X., Xiang Y., Bertino E., Zhou J., Xu L. (2013). Robust multi-factor authentication for fragile communications. IEEE Trans. Depend. Secur. Comput..

[28] Wang D., He D., Wang P., Chu C. (2015). Anonymous two-factor authentication in distributed systems: Certain goals are beyond attainment. IEEE Trans. Depend. Secur. Comput..

[29] Jiang Q., Zeadally S., Ma J., He D. (2017). Lightweight three-factor authentication and key agreement protocol for internet-integrated wireless sensor networks. IEEE Access.

[30] Wang D., Wang P. (2016). Two birds with one stone: Two-factor authentication with security beyond conventional bound. IEEE Trans. Depend. Secur. Comput..

[31] Kumari S., Li X., Wu F., Das A.K., Choo K.K.R., Shen J. (2017). Design of a provably secure biometrics-based multi-cloud-server authentication scheme. Futur. Gener. Comput. Syst..

[32] He D., Wang D. (2015). Robust biometrics-based authentication scheme for multiserver environment. IEEE Syst. J..

[33] Jiang Q., Chen Z., Li B., Shen J., Yang L., Ma J. (2017). Security analysis and improvement of bio-hashing based three-factor authentication scheme for telecare medical information systems. J. Ambient Intell. Humaniz. Comput..

[34] Lee C.C., Chen C.T., Wu P.H., Chen T.Y. (2013). Three-factor control protocol based on elliptic curve cryptosystem for universal serial bus mass storage devices. IET Comput. Digit. Tech..

[35] He D., Zeadally S., Kummar N., Wu W. (2016). Efficient and anonymous mobile user authentication protocol using self-certified public key cryptography for multi-server architectures. IEEE Trans. Inf. Forensics Secur..

[36] Wang C., Wang D., Xu G., Guo Y. (2017). A lightweight password-based authentication protocol using smart card. Int. J. Commun. Syst..

[37] Wang D., Cheng H., Wang P., Huang X., Jian G. (2017). Zipf’s law in passwords. IEEE Trans. Inf. Forensics Secur..

[38] Kumari S., Khan M.K., Li X., Wu F. (2016). Design of a user anonymous password authentication scheme without smart card. Int. J. Commun. Syst..

[39] Li X., Qiu W., Zheng D., Chen K., Li J. (2010). Anonymity enhancement on robust and efficient password-authenticated key agreement using smart cards. IEEE Trans. Ind. Electron..

[40] Li X., Xiong Y., Ma J., Wang W. (2012). An enhanced and security dynamic identity based authentication protocol for multi-server architecture using smart cards. J. Netw. Comput. Appl..

[41] Ma1 C., Wang D., Zhao S. (2012). Security flaws in two improved remote user authentication schemes using smart cards. Int. J. Commun. Syst..

[42] Wang C., Xu G. (2017). Cryptanalysis of three password-based remote user authentication schemes with non-tamper-resistant smart card. Secur. Commun. Netw..

[43] Burrows M., Abadi M., Needham R. (1990). A logic of authentication. IEEE Trans. Comput..

[44] Amin R., Islam S.H., Biswas G.P., Khan M.K., Leng L., Kumar N. (2016). Design of an anonymity-preserving three-factor authenticated key exchange protocol for wireless sensor networks. Comput. Netw..

